# Advances in the Health, Behavior, and Physiology of Honeybees and Other Pollinators

**DOI:** 10.3390/insects17020231

**Published:** 2026-02-23

**Authors:** Giovanni Formato, Franco Mutinelli

**Affiliations:** 1 FAO RC for Apiculture: Health, Biosecurity and AMR, WOAH Collaborating Centre for Good Beekeeping Management Practices and Biosecurity Measures in the Apiculture Sector, Istituto Zooprofilattico Sperimentale del Lazio e della Toscana, Via Appia Nuova 1411, 00178 Rome, Italy; giovanni.formato@izslt.it; 2 NRL for Honey Bee Health, NRL for Honey Bee Health and FAO RC for Apiculture: Health, Biosecurity and AMR, Istituto Zooprofilattico Sperimentale delle Venezie, Viale dell’Università 10, 35020 Legnaro, Italy

The health, behavior, and physiology of honeybees and other pollinators are critical areas of research with profound implications for biodiversity, agriculture, and ecosystem stability. As keystone species, pollinators are essential for the reproduction of many plants, influencing both natural and agricultural ecosystems. Recent scientific advancements have highlighted the complex interplay of factors affecting pollinator health, including environmental stressors, pathogens, pesticides, and habitat loss. Understanding these dynamics is vital for developing strategies to mitigate their decline and ensure ecological and agricultural sustainability.

This Special Issue of *Insects* on “Advances in the Health, Behavior, and Physiology of Honeybees and Other Pollinators” aims to consolidate cutting-edge research in this pivotal field. Aligning with the journal’s focus on the biology, ecology, and management of insects, this Special Issue will explore innovative findings and foster interdisciplinary discourse, and researchers are invited to submit original research articles, reviews, and case studies that contribute to our understanding of pollinator health and behavior, their physiological responses to various environmental pressures, and the development of conservation strategies.

Topics of interest include, but are not limited to, the impact of pesticides and pathogens, the effects of climate change, habitat requirements, genetic diversity, current and innovative management practices, and bee welfare. We look forward to receiving contributions that will drive forward the scientific understanding of and conservation efforts for these indispensable insects.

The Special Issue “Advances in the Health, Behavior, and Physiology of Honeybees and Other Pollinators” has published 10 papers, including 8 research articles, 2 review articles, and 1 communication article. This Editorial explores how beekeeping practices, parasite control measures, and exposure to toxic substances influence the health and survival of honey bee colonies.

Al-Ghzawi et al. [Contribution 1] evaluated the effects of preheat hardening on honeybee queen egg-laying and the flight performance of their daughter workers. Eggs from caged queens were incubated at either 41 °C (pre-heat-treated queens, pH-TQs, *n* = 12) or 34.5 °C (non-heat-treated queens, nH-TQs, *n* = 12) for 15 min. After mating, both groups were introduced into queenless hives. Colonies headed by pH-TQs reared significantly more brood and adult workers—especially in summer—collected more pollen, and produced fewer drone brood and swarm cells than nH-TQ colonies. These results suggest preheat hardening enhances reproductive potential and adaptability in honeybees, offering a practical approach for improving colony performance and resilience in the face of global honeybee declines.

Self-regulatory foraging in honey bees (*Apis mellifera*) was examined by Jones et al. [Contribution 2] using Perceptual Control Theory (PCT). We developed a PCT-based model to describe how bees maintain goal-directed behavior toward a sucrose-rich feeding site while overcoming wind disturbances. In controlled experiments, 13 out of 14 bees successfully adjusted flight paths to reach the target, showing substantial individual variability in strategies. Ultimately, the bees consistently adopted a headwind approach rather than tailwind or crosswind. These findings support the view that honey bee foraging is self-regulative—dynamic, non-linear, and goal-directed—rather than a simple input–output sequence. PCT provides a framework to model such teleonomic behaviors, emphasizing functional purpose in behavioral regulation.

The increasing demand for honey bee (*Apis mellifera*) pollination has intensified efforts to preserve genetic diversity and support breeding programs. Cryopreservation of drone semen offers a solution without the risks associated with live animal transport. Egyptien et al. [Contribution 3] evaluated a one-step, antibiotic-free slow-freezing protocol under field conditions using in vitro and in vivo assessments. Post-thaw semen viability decreased by 37%, yet virgin queens inseminated with frozen–thawed semen produced brood at rates comparable to those inseminated with fresh semen, although winter survival was higher in the fresh semen group. These findings indicate that frozen–thawed semen can support viable queen insemination and brood production, highlighting the potential for cryobanking while emphasizing the need for protocol refinement to enhance semen quality and queen longevity.

Honeybee combs are central to colony development, serving as sites for brood rearing, food storage, and other essential activities. As combs age through successive brood cycles, their structural and physicochemical properties deteriorate, negatively affecting colony health and productivity. This review by Meng et al. [Contribution 4] synthesizes current knowledge on comb functions, aging processes, and associated impacts, including altered cell dimensions, smaller worker bees, reduced foraging efficiency, and compromised honey and beeswax quality. Honeybees mitigate these effects through hygienic behaviors, comb rebuilding, and enhanced immune and detoxification responses. Systematic replacement of old brood combs is emphasized as a key management practice to maintain colony strength, optimize bee health, and support sustainable apiculture.

Honey bees (*Apis mellifera*) are globally important pollinators and economically valuable insects, yet they face numerous stressors that threaten colony health. Adequate nutrition is critical for maintaining strong, resilient colonies, requiring balanced intake of proteins, carbohydrates, lipids, amino acids, vitamins, minerals, water, and essential sterols, with multi-floral pollen being ideal. Colonies in low-diversity agroecosystems often experience reduced brood rearing and longevity, increasing susceptibility to parasites and diseases. Nutritional supplements have been developed to support colony health, but nutrient deficiencies may persist. In this review, Ansaloni et al. [Contribution 5] highlight the relationship between dietary requirements and colony well-being, emphasizing the need for supplements that better mimic natural diets and future research to evaluate long-term impacts on colony resilience, particularly in resource-limited environments.

Honey bee health and productivity are strongly shaped by management and biosecurity practices. Gratzer et al. [Contribution 6] analyzed 744 records from 191 field studies (1995–present), categorizing interventions as good beekeeping practices (17.2%) or biosecurity measures (82.8%) across management themes, pathogens, regions, and seasons. Most studies focused on *Apis mellifera* in Europe (34.6%) and North America (33.4%). Varroa control dominated (57.0%), primarily through using soft acaricides (58.5%), while general colony management (17.2%) and American foulbrood interventions (antibiotics 41.7%) were also common. Seasonal trends showed most interventions occurred from August to October, with foulbrood peaks in spring–early summer. This dataset highlights regional research gaps and provides a structured framework to guide evidence-based decision-making in beekeeping.

*Varroa destructor* is a key driver of global honey bee colony declines, and miticide applications are widely used to control infestations. While forager exposure has been studied extensively, nurse bees are also vulnerable due to direct contact and residual hive exposure. Kim et al. [Contribution 7] assessed the toxicities of single and consecutive applications of three synthetic miticides (fluvalinate, coumaphos, amitraz) and two organic miticides (formic acid, oxalic acid) at field-realistic doses. Synthetic miticides caused minimal mortality, whereas organic miticides—particularly formic acid—significantly reduced survivorship. Consecutive treatments largely mirrored the toxicity of the organic agent alone, with the fluvalinate–formic acid combination showing no significant adverse effects. These results emphasize careful miticide use and inform safer strategies to protect in-hive nurse bees.

The small hive beetle (*Aethina tumida*) threatens honeybee (*Apis mellifera*) populations in Europe, with potential spread from Calabria, Italy. Early detection is critical, as current methods often fail, leading to colony losses. Ghisellini et al. [Contribution 8] developed a biosensor to detect *Kodamaea ohmeri*, a yeast symbiotically associated with *A. tumida*, in honey samples. Using a quartz crystal microbalance with a gold-plated surface functionalized with a specific antibody, the biosensor identified peptides linked to *K. ohmeri*, offering a potential early-warning system for infestations. The preliminary results demonstrate the feasibility of this approach to support rapid and targeted colony management. Further miniaturization, cost reduction, and beekeeper training are recommended to enhance adoption and improve honeybee health and productivity.

Lambda-cyhalothrin (LCY), a widely used pyrethroid insecticide, is toxic to honey bees, yet its molecular effects during early development are poorly understood. Vasamsetti et al. [Contribution 9] examined chronic sublethal LCY exposure in larvae and adults, comparing solvent-treated and LCY-treated groups. Differential gene expression analysis revealed 1128 altered genes in larvae and 168 in adults, with stage-specific effects on 125 larval and 25 adult genes. LCY disrupted cuticle formation, sulfur metabolism, oxidoreductase activity, and neuropeptide signaling in larvae, while affecting redox balance, peptide receptor signaling, and monoamine transport in adults. Both stages showed neuroactive signaling disruptions, with larvae additionally showing impaired motor function, amino acid metabolism, and glycolysis. Downregulation of chitin metabolism and antioxidant genes in larvae suggests weakened exoskeletal integrity. These findings highlight lasting molecular consequences of early-life LCY exposure and underscore the need for safer pesticide practices to protect pollinators.

Honey bees (*Apis mellifera*) are critical pollinators, yet pesticide exposure poses risks to their health. Hu et al. [Contribution 10] investigated the sublethal effects of atrazine on honey bee sucrose sensitivity and the underlying molecular mechanisms via transcriptomic analysis. Atrazine exposure significantly reduced sucrose sensitivity, likely by suppressing brain genes involved in cognition. Differentially expressed genes were associated with neurodegenerative processes and behavioral regulation, indicating that atrazine can disrupt neural function and foraging behavior. These findings provide new insights into the neurophysiological and behavioral impacts of atrazine, highlighting potential risks to pollinator health and informing strategies for safer pesticide use and environmental regulation.

This Special Issue, “Advances in the Health, Behavior, and Physiology of Honeybees and Other Pollinators”, brings together a diverse and relevant collection of contributions addressing key challenges and emerging knowledge in honey bee science. The ten papers published in this Special Issue, including original research articles, review articles, and a communication, highlight the interdisciplinary nature of current research in apiculture and pollinator biology.

Pollinators continue to face unprecedented pressures from pathogens, environmental change, habitat loss, and anthropogenic stressors. At the same time, advances in physiology, behavior, diagnostics, biosecurity measures, and management practices are expanding our ability to understand and protect these essential animals. The studies presented in this Special Issue reflect both scientific rigor and practical relevance, bridging fundamental research with applied perspectives that are critical for sustainable beekeeping and ecosystem resilience.

We hope that this compilation will stimulate further research, foster international collaboration, and contribute to evidence-based strategies for protecting honeybees and other pollinators. Continued investment in pollinator science is not only a scientific priority but also a societal necessity, given the central role of pollinators in food security, biodiversity, and ecosystem services.

We trust that readers will find inspiration in these contributions and that this collection will serve as a foundation for future advances in pollinator research.

## Data Availability

Data can be requested to the authors of the contributions collected in this SI.

